# The Extraordinarily Complex but Highly Structured Organization of Intestinal Mucus-Gel Unveiled in Multicolor Images

**DOI:** 10.1371/journal.pone.0018761

**Published:** 2011-04-12

**Authors:** Valérie Gouyer, Frédéric Gottrand, Jean-Luc Desseyn

**Affiliations:** Inserm U995, Université Lille Nord de France, Lille, France; The University of Kansas Medical Center, United States of America

## Abstract

The mucus that coats the gastrointestinal tract of all mammals is a dynamic and sticky gel layer and represents the first protective barrier between the host and the hostile environment. There is, however, a lack of detailed knowledge about the mucus gel organization because of the high water content and the complexity of MUC2, the main gel-forming molecule in the intestine. Histological staining and a multilabel immunofluorescence method were used to examine mucus blankets and Muc2 in mouse colon and ileum samples fixed in Carnoy's solution, unveiling an extraordinarily complex but highly structured mucus gel organization. The inner firmly adherent mucus blanket consists of alternating layers. The thicker outer loosely adherent mucus blanket in the colon is made of alternating laminated layers and loose curl-like structures. The layers consist of Muc2 molecules with different fucosylation states and glycoforms remain unmixed in the mucus. Importantly, distinct goblet cell subpopulations throughout the ileum along the crypt-to-villus axis with an alternation of goblet cells secreting fucosylated and non-fucosylated Muc2 are observed. A better understanding of the mucus structure should contribute to improve the efficiency of DNA and drug delivery and will allow for a better understanding and treatment of inflammatory and infectious intestinal diseases.

## Introduction

The gastrointestinal mucus is an essential complex gel-like secretion that protects the stomach and duodenum against acidic juice, prevents tissue dehydration, lubricates the gastrointestinal tract for the movement of foodstuffs and represents the first innate line of defense against the hostile environment. Its physical properties are dictated mainly by secreted gel-forming mucin MUC2 molecules [1]. However, the membrane-bound mucins (ie Muc1, Muc4, Muc17/mouse Muc3, Muc15) may likely play a role in the physical properties of mucus gels. They are located primarily, but not exclusively, at the cell surface, as their respective genes may encode splicing variants for secreted proteins or/and as the heavily *O*-glycosylated extracellular portion may be released into the mucus-gel by proteolysis (for review see [2]). Muc2-null mice demonstrated the key role of the mucin in the epithelial defense [3]–[6]. MUC2 is large and extensively *O*-glycosylated and secreted by goblet cells as polymers [4], [7] to form a firmly adherent inner gel layer adjacent to the underlying epithelium and, on the top of this, a loosely adherent outer layer [8]. A gradation of the *O*-glycosylation along the crypt-to-villus (or crypt-to-surface epithelial cuff) axis has been observed [9], [10]. However, the behavior of each mucin glycoform after its discharge from goblet cells into the lumen is largely unknown. In the colon, it is believed that the fibrous-appearing layers observed either in the outer [11] or in the inner [4] mucus layer, depending on the study, arise from the diversity of MUC2 glycoforms. In fact, the high water content of mucus and the complex structure of mucins have contributed to our lack of knowledge of mucus organization. However, common histological staining and multilabel immunofluorescence used on mouse samples fixed with Carnoy's fixative are very valuable techniques as they revealed for the first time the extraordinarily complex but highly structured organization of the intestinal mucus-gel.

## Materials and Methods

### Tissue samples

Animal tissues were obtained from 6-wk-old C57BL/6 mice (Charles River, France) that were housed in microisolator cages in a specific pathogen-free animal facility and maintained on pelleted rodent chow and water ad *libitum*. Animals were euthanized by cervical dislocation. All the experiments were conducted in agreement with guidelines and the Animal Care Committee.

### Histology and immunohistochemistry

Because of the high water content of mucus, tissues were fixed in Carnoy's solution, which provides the most satisfactory preservation of the mucus layers in paraffin blocks [11], [12], then embedded in paraffin. Five-micron-thick sections were prepared. Alcian Blue–periodic acid Schiff (AB–PAS) staining and anti-MUC2 staining were performed as previously described [13] using H300 anti-MUC2 polyclonal antibody (Santa Cruz). The lectin *Ulex europaeus* agglutinin I (UEA1), which recognizes H type 2 epitopes (Fuc α-1,2-Gal) and the lectin *Maackia amurensis* agglutinin (MAA), which recognizes the epitope sialic acid α-2,3-galactose, conjugated directly to TRITC (EY laboratories, CA) were used at 25 µg/ml. Sections used for immunofluorescence experiments were incubated with Hoechst 33258 (1∶1000) to stain nuclei. Images are representative of 10 fields randomly chosen per colon and per ileum using 8 C57BL/6 mice.

### Imaging

Multilabel immunofluorescence analysis was performed on a Leica DM4000B or a Zeiss LSM 710 confocal microscope. Images were acquired and minimally processed by importing them into the GNU image manipulation program GIMP.

## Results and Discussion

**Figure 1:**
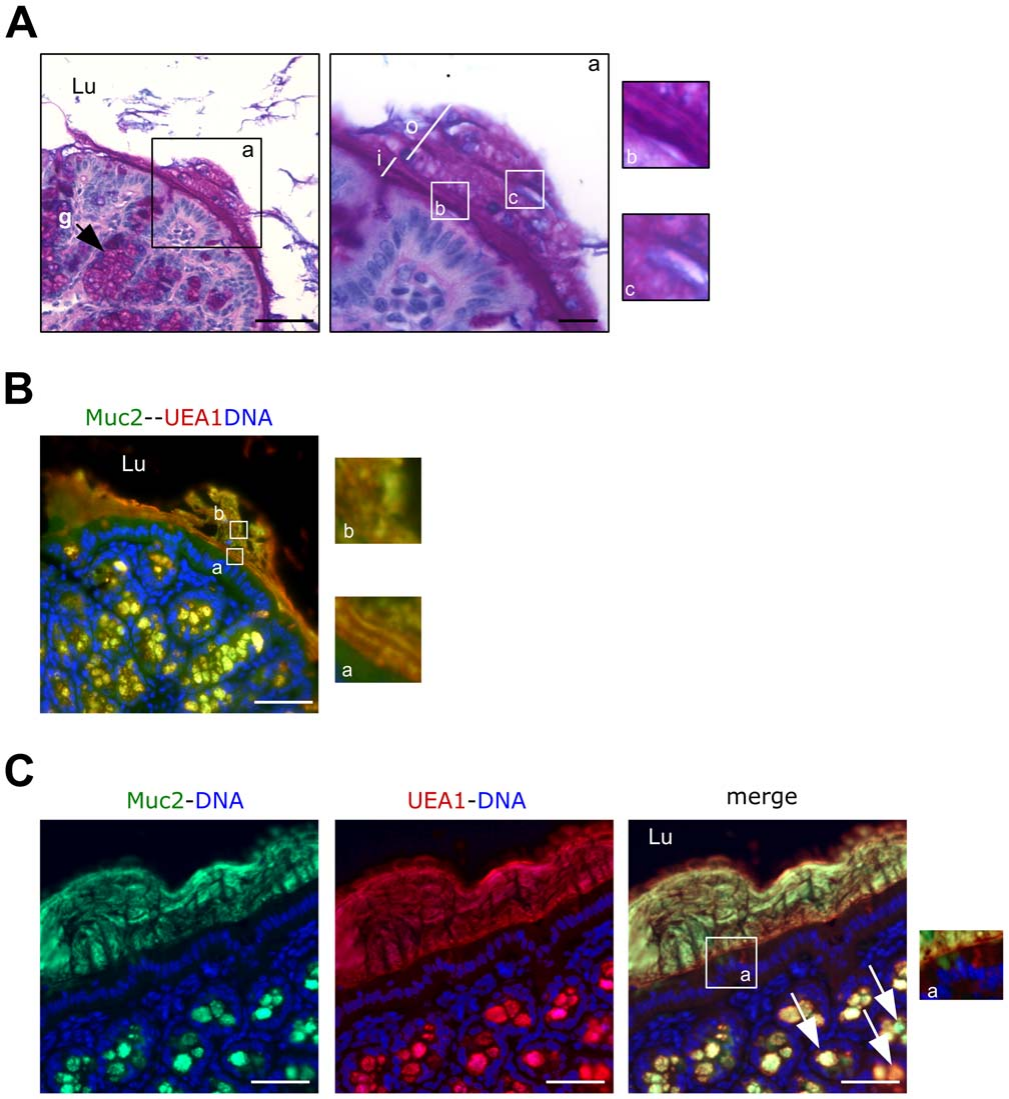
Multilabel immunohistochemical analysis of Muc2 in the mouse colon. (**A**) AB–PAS staining of a paraffin-embedded mouse colon section showing the inner (i) and the outer (o) mucus blankets. The inner layer exhibits a horizontal stratified arrangement while the outer layer, which is more difficult to preserve and is partly detached, is thicker than the inner layer and shows an alternation of horizontal laminated multilayered structures and loose curl-like structures. g: goblet cell. (**B**) and (**C**) Immunofluorescence analysis of Muc2 and UEA1. Only the inner mucus blanket is preserved in C. Muc2 was visualized using an anti-MUC2 antibody (green) that recognizes both human and mouse Muc2 and UEA1 lectin (red) that recognizes H-type 2 epitopes. Goblet cells (arrows) discharge Muc2 molecules that do or do not carry H type 2 epitopes demonstrating that UEA1-negative mucin does not result from degradation of UEA1-positive Muc2 polymers by bacteria. All UEA1-positive goblet cells (red) are Muc2 positive while not all Muc2 positive goblet cells are UEA1 positive. The high power in C shows exocytosis of Muc2 with no H type 2 epitopes (green) from a goblet cell close to another goblet cell that is discharging Muc2 carrying H type 2 epitopes (red) into the lumen. The inner mucus blanket is made up of multiple unmixed horizontal layers of mucin flanked by the most fucosylated layers. Bars  =  50 µm. Lu: lumen.

Histological staining and immunofluorescence imaging of mouse biopsies using an anti-MUC2 antibody in combination with lectins offers for the first time an unprecedented glimpse into the well-organized mucus layer of the gastrointestinal tract. Using AB–PAS staining, the colonic inner mucus blanket, which is attached more consistently to the apical surface of the epithelial cells than the outer mucus blanket, consisted of a tight, multilayered structure, while the thick, weakly adherent outer mucus blanket, which is more difficult to preserve, was made of alternating laminated layers and loose curl-like structures (Fig. 1A). A method to visualize the two colonic mucus layers in a mucus-preserving conditions has been described before (see [14] and therein) but this technique used snap-frozen tissue and cryostat cross-sections and does not allow cytological detail to be studied.

Fucosylated and sulfated oligosaccharides are abundant in the carbohydrate moieties of the mouse Muc2 [15] and these Muc2 glycoforms may contribute to the gel organization. Immunofluorescence images showed an inner mucus blanket consisting of alternating layers consisting of Muc2 molecules with different fucosylation states. Remarkably, the most fucosylated glycoforms enveloped the inner mucus blanket (Fig. 1B and Fig. 1C). The diversity of glycoforms in the colon arose at least partly from different goblet cell subpopulations, because some goblet cells produced glycoproteins (mucins) carrying H type 2 epitopes while others did not (Fig. 1C). Goblet cell subpopulations have been previously observed in the human colonic epithelium using antibodies raised against purified colonic mucin [16]. We were also able to visualize a goblet cell exocytosis where the discharged Muc2 molecules carried H type 2 epitopes next to another goblet cell discharging nonfucosylated Muc2 molecules (Fig. 1C). Staining with MAA lectin did not reveal the goblet cell subtypes or the alternation of mucus layers in the colon (data not shown). The stratification of mucin layers was previously reported in stomach where the two gel-forming mucins MUC5AC and MUC6 remained segregated within the mucous gel in a laminated linear arrangement [17].

**Figure 2:**
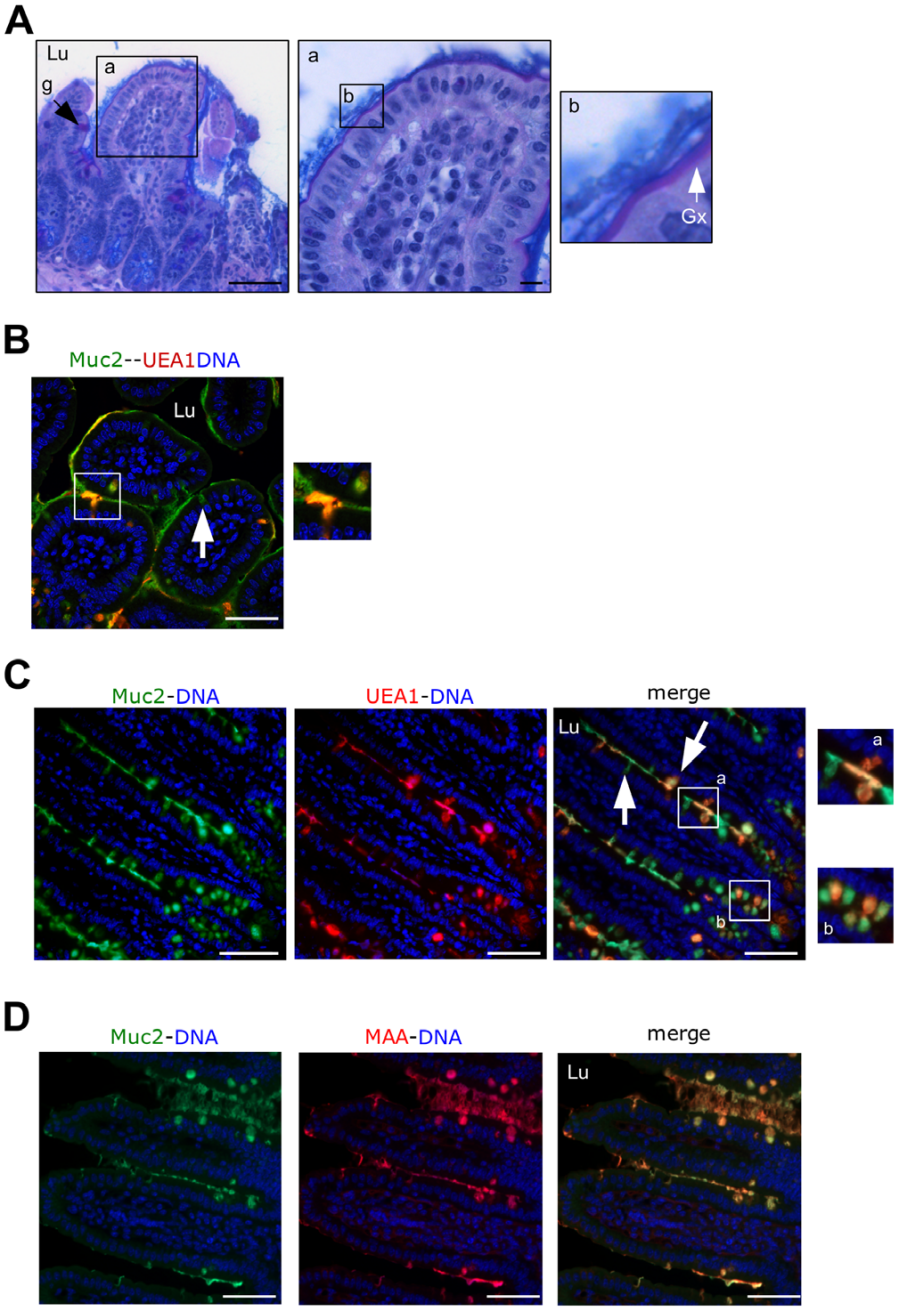
Alternation of goblet cell types in the mouse ileum producing unmixed Muc2 glycoforms. (**A**) AB–PAS-stained section from a mouse ileum showing the glycocalyx (purple) and the mucus gel between villi and at the top of the villi. Gx: glycocalyx; g: goblet cell. (**B–C**) Ileum section stained as in Fig. 1. Nonfucosylated and fucosylated Muc2 mucin polymers secreted along villi (high power Ba) and within crypts (high power Bb) form a long filamentous sticky rope-like structure made of unmixed Muc2 glycoforms. Arrows outline the diversity of goblet cells depending of the fucosylation level of Muc2. (**D**) Colocalisation of Muc2 (green) and MAA (red) which recognized the sialic acid α-2,3-galactose epitope. No goblet cell subpopulations were observed. Bars  =  50 µm. Lu: lumen.

Histological analysis of mouse ileum using AB–PAS showed a mix of neutral and acidic mucins in goblet cells and in the mucus, with a compact mucus blanket coating the mucosa and a loose mucus layer between villi (Fig. 2A). Importantly, imaging using the fucose-specific lectin UEA1 and anti-MUC2 antibody disclosed distinct goblet cell subpopulations throughout the ileum along the crypt-to-villus axis with an alternation of two goblet cell subpopulations (Fig. 2B and high power inset). The heterogeneity of goblet cells previously noticed by others in crypts of the rat small bowel using antibodies raised against mucin from the rat intestine [10] was also visible in the crypts (Fig. 2B). Muc2 glycoforms formed a long, sticky, rope-like structure that seemed to flow toward the top of the villi. Importantly, the glycoforms remained unmixed in an unforeseen manner (Fig. 2C). Sialic acid-specific lectin MAA could not distinguish between goblet cell subtypes (Fig. 2D).

Mucus gel is the first innate line of defense that also acts as an efficient semipermeable barrier in the digestive tract. This dynamic, sticky, insulating blanket made of many layers with unique, distinct biochemical properties is highly structured. A better understanding of mucus architecture should help to understand and prevent pathogen entry, to improve the engineering of particle-based systems to better penetrate the mucus layer for drug delivery and gene therapy and, alternatively, to permit reinforcement of its properties against pathogens and chemical substances.
